# Testosterone Reduces Growth and Hepatic *IGF-1* mRNA in a Female-Larger Lizard, *Sceloporus undulatus*: Evidence of an Evolutionary Reversal in Growth Regulation

**DOI:** 10.1093/iob/obaa036

**Published:** 2020-10-28

**Authors:** Christine A Duncan, Wendie S Cohick, Henry B John-Alder

**Affiliations:** Graduate Program in Endocrinology and Animal Biosciences, Rutgers University, 84 Lipman Drive, New Brunswick, NJ 08901, USA; Graduate Program in Endocrinology and Animal Biosciences, Rutgers University, 84 Lipman Drive, New Brunswick, NJ 08901, USA; Graduate Program in Endocrinology and Animal Biosciences, Rutgers University, 84 Lipman Drive, New Brunswick, NJ 08901, USA; Department of Ecology, Evolution, and Natural Resources, Rutgers University, 14 College Farm Road, New Brunswick, NJ 08901, USA

## Abstract

Previous research has demonstrated that testosterone (T) can inhibit growth in female-larger species and stimulate growth in male-larger species, but the underlying mechanisms of this regulatory bipotentiality have not been investigated. In this study, we investigated the effects of T on the expression of hepatic insulin-like growth factor-1 (*IGF-1*) mRNA and circulating IGF-1 hormone in *Sceloporus undulatus*, a species of lizard in which females grow faster to become larger than males and in which T inhibits growth. Experiments were performed in captivity on mature female and male adults in the asymptotic phase of their growth curve and on actively growing, pre-reproductive juveniles. In adult males, the expression of hepatic *IGF-1* mRNA increased following surgical castration and returned to control levels with T replacement; in intact adult females, exogenous T had no effect on *IGF-1* mRNA expression. In juveniles, T significantly reduced both growth and the expression of hepatic *IGF-1* mRNA to similar extents in intact females and in castrated males. The relative inhibitory effects of T on mRNA expression were greater in juveniles than in adults. Plasma IGF-1 hormone was about four times higher in juveniles than in adults, but T had no significant effect on IGF-1 hormone in either sex or in either age group. Our finding of inhibition of the expression of hepatic *IGF-1* mRNA stands in contrast to the stimulatory effects of T in the published body of literature. We attribute our novel finding to our use of a species in which T inhibits rather than stimulates growth. Our findings begin to explain how T has the regulatory bipotentiality to be stimulatory in some species and inhibitory in others, requiring only an evolutionary reversal in the molecular regulation of growth-regulatory genes including *IGF-1*. Further comparative transcriptomic studies will be required to fully resolve the molecular mechanism of growth inhibition.

## Introduction

Body size is one of the most obvious traits of any organism, but its ease of measurement belies its pervasive biological importance (Peters 1983; Dunham and Miles 1985; Calder 1996) and developmental complexity (Schreibman et al. 1993, Nijhout et al. 2010). The importance of body size can be inferred from the widespread observation that even within species, females and males have evolved different optimal body sizes (i.e., sexual size dimorphism [SSD]; Fairbairn et al. 2007) accompanied by sex differences in growth (Manicom et al. 2014). In squamate reptiles (lizards and snakes), for example, either females or males can be as much as 50% larger (in length) than the other sex (Cox et al. 2003, 2007), and in some cases, opposite patterns of SSD have evolved even among closely related species (e.g., Cox and John-Alder 2007). In common with other sexual dimorphisms (Williams and Carroll 2009), sex differences in growth and body size exist even though females and males of a species share nearly identical genomes (Lande 1980; Chenoweth et al. 2008), and this focuses attention on sex differences in gene expression (Badyaev 2002; Mank 2009) as the predominant cause of the development of sex differences in body size. This study investigates how testosterone (T) regulates the expression of a key growth-controlling gene in a female-larger, sexually size dimorphic lizard.

As reviewed by Williams and Carrol (2009), gonadal steroids mediate the development of most sexual dimorphisms in vertebrates. This has specifically been found to be the case for sex-specific growth in sexually size dimorphic vertebrates. In some Iguanian lizards, for example, SSD develops because of sex differences in growth rate that are temporally associated with sexual divergence in plasma T (Cox et al. 2005; Cox and John-Alder 2005). Experimental administration of T has the bipotential capacity to either stimulate or inhibit growth in these species (Cox and John-Alder 2005; John-Alder et al. 2007), where the directional effects of T are concordant with each species’ natural pattern of SSD (Cox et al. 2009). In several Gekkotan species of lizards, ovarian hormones (especially estrogens) may be more important than T in the development of SSD (see Kubička et al. 2017). In this regard, sex-specific growth regulation in squamate reptiles is reminiscent of the situation in fishes, in which androgens appear to be the dominant growth-regulatory steroids in most species, while estrogens may be dominant in others (Goetz et al. 2009). Furthermore, even while T stimulates growth and contributes to the development of SSD in *Anolis sagrei* (Cox et al. 2015), estrogens rather than androgens may underlie the development of cranial sexual dimorphism in this and other species of *Anolis* (Sanger et al. 2014). Given that SSD is a manifestation of the body sizes of males compared to females, its development is almost certainly influenced by both androgenic and estrogenic gonadal steroids.

The underlying mechanism(s) of steroidal growth regulation in lizards—including bipotential effects of T—are unresolved (John-Alder et al. 2007), but reports on other classes of vertebrates (Borski et al. 1996) and a recent report on *A. sagrei* (Cox et al. 2017) implicate the somatotrophic axis, also known as the growth hormone (GH) / insulin-like growth factor-1 (IGF-1) axis (i.e., central endocrine growth axis), as a fruitful starting point for investigation. Vertebrate growth is primarily mediated by the somatotrophic axis, in which pituitary growth hormone (GH) binds hepatic receptors and stimulates the synthesis and secretion of hepatic (systemic endocrine) IGF-1, which then promotes cell proliferation and growth in target tissues (Le Roith et al. 2001; Wood et al. 2005; Laviola et al. 2007). Activity of the GH/IGF-1 axis is influenced by energy, nutrients, and numerous hormones including sex steroids (Reindl and Sheridan 2012), suggesting that sex steroids operate through the somatotrophic axis to produce sex differences in growth (Gatford et al. 1998). In most species that have been investigated to date, growth-stimulatory effects of T are typically associated with increases in the expression of hepatic *IGF-1* mRNA and plasma levels of IGF-1 hormone (Borski et al. 1996; Riley et al. 2002; Larsen et al. 2004; Beckman 2011; Cox et al. 2017), while estradiol (E_2_) has the opposite effect (Murphy and Friesen 1988; Hanson et al. 2014). Even within the same species, functional activity of the somatotrophic axis can be increased by T and decreased by E_2_, not only in whole organisms but also in primary hepatic cell culture (Riley et al. 2004; Norbeck and Sheridan 2011). In other species, however, E_2_ stimulates growth and may increase the expression of hepatic *IGF-1* mRNA (Goetz et al. 2009), suggesting evolutionary lability in the molecular identity of the “on” switch for the growth-regulatory network.

How, then, can an identical signal, T, have the bipotential capacity to stimulate growth in male-larger species while inhibiting growth in female-larger species of lizards? In this study, we investigate the underlying mechanism of T-induced growth inhibition in Eastern fence lizards (*Sceloporus undulatus*), a species in which females grow faster to become larger than males (Haenel and John-Alder 2002) and in which T has previously been shown to inhibit growth (Cox et al. 2005). Given the weight of previously published evidence, the stimulatory effect of T on the expression of hepatic *IGF-1* mRNA may have been evolutionarily conserved in both male- and female-larger species, and atypical growth inhibition by T may then reflect upstream or downstream mechanisms to negate the growth-promoting effect of T in female-larger species. In other words, a tight linkage between T and its phenotypic effects may have been evolutionarily conserved, consistent with the evolutionary constraint hypothesis (Hau 2007), and other mechanisms would have to be invoked to prevent the stimulation of growth. Alternatively, fundamentally different effects of T on the expression of hepatic *IGF-1* mRNA may have evolved in female-larger versus male-larger species, consistent with the evolutionary potential hypothesis (Hau 2007), which allows flexibility in the linkage between a hormone and its effects. The plausibility of this latter hypothesis is strengthened by the report that T differentially regulates gene expression even between sexes of a single species, which indicates that differences in responses to common hormonal regulators can evolve readily. In the experiments reported here, we tested this hypothesis by investigating the predictions that T will reduce growth as well as the expression of hepatic *IGF-1* mRNA and levels of plasma IGF-1 peptide in *S. undulatus*. Our study represents a specific test of evolutionary lability in the effects of T on the central vertebrate growth-regulatory network (see Hau 2007; McGlothlin and Ketterson 2008; Cox 2020).

## Material and methods

### Animal collection and care

In June 2009, adult males and females (≥2 years of age; minimum snout–vent length [SVL] 56 mm) of *S. undulatus* were captured by hand in the pinelands of New Jersey, USA (41°N, 74°35′W) under permit from the New Jersey Department of Environmental Protection, Division of Fish and Wildlife (permit #SC 2909C). In September 2009, juvenile males and females (maximum SVL 40 mm measured on October 16) of *S. undulatus* were collected from the same field site. Age classes were established on the basis of published (Haenel and John-Alder 2002) and unpublished demographic records of *S. undulatus* in the NJ Pinelands, and the narrow age estimate of juveniles was based on records of HJ-A regarding the first appearance of hatchlings, typically around the beginning of August. All lizards were subsequently transported to and housed under permit in our laboratory facility at Rutgers University (permit #SH 29097). Adults were maintained for 6–27 days before experiments were begun. Juveniles were allowed to grow in captivity for 4 months prior to experimentation, until they were exhibiting indicators of sexual maturation (e.g., sex-specific pigmentation) and were large enough for easy surgical manipulations. All lizards were housed individually in plastic cages (59.1 × 43.2 × 45.7 cm^3^) with a 2-cm substrate of granular bedding (Marcal^®^ KaoBed^™^) and two concrete bricks, which were stacked under the heat source to create shelter and provide a basking site. An incandescent spotlight (Philips 65 W BR-40SP, Royal Philips Electronics, Netherlands) was suspended above the bricks to provide a 10 h basking period. Full-spectrum fluorescent bulbs (General Electric Chroma 50, General Electric Company, Fairfield, CT) were hung ∼60 cm above the floor of the cage to provide a 12:12 light:dark photoperiod. Water was provided *ad libitum* in a shallow dish lined with aquarium gravel. Cages were separated by opaque dividers to prevent visual interactions. All experiments were approved by the Rutgers University Animal Care and Facilities Committee (protocol number 01-019).

### Experimental design: Growth and feeding

Immediately prior to surgical manipulation, SVL was measured to the nearest 0.5 mm and body mass was measured to the nearest 0.02 g. These data were used to rank lizards by size and to assign them to size-matched treatment groups. During experiments, SVL and body mass were measured weekly (juveniles) or bi-weekly (adults) to support calculations of growth rate. Growth rate (mm/day) was calculated by dividing change in length by number of days for each interval that was analyzed, including the 14-day experimental period for adults and three growth intervals for juveniles (two pre-treatment intervals plus the experimental period). For the purposes of these calculations, we assumed linear growth within each interval. For juveniles, the first pre-treatment growth interval was 35 days, the second was 59 days, and the experimental interval was 36 days. *A priori*, we did not expect to be able to detect growth in the adults because these individuals were on the asymptotic part of their growth curve (Haenel and John-Alder 2002; John-Alder et al. 2007) and because the duration of the experiment was only 14 days (see below).

Throughout their time in captivity, both before and during their experimental periods, lizards were fed three crickets per day. Food consumption was measured by counting uneaten crickets every 3 days (adults) or every 7 days (juveniles). Adults were fed larger crickets than juveniles, rendering comparisons of food consumption valid within but not between the age classes of the two experiments (see below).

#### Experiment 1: Adults

Adults were divided into five size-matched treatment groups: (1) sham-surgery + placebo-implanted control males (M-CON; *n* = 9; SVL: 64 ± 1.2 mm; range 58–70 mm); (2) surgically castrated + placebo-implanted males (M-CAST; *n* = 9; SVL: 64 ± 1.2 mm; range 56–68 mm); (3) surgically castrated + T-implanted males (M-TEST; *n* = 9; SVL: 63 ± 1.0 mm; range 57–67 mm); (4) placebo-implanted control females (F-CON; *n* = 9; SVL: 67 ± 1.4 mm; range 60–72 mm); and (5) T-implanted females (F-TEST; *n* = 9; SVL: 68 ± 1.4 mm; range 61–75 mm). The 14-day treatment period began immediately following surgery.

#### Experiment 2: Juveniles

At the time of capture, SVLs of juvenile males and females were statistically indistinguishable (♂ 36.5 ± 0.47 vs. ♀ 37.2 ± 0.30 mm; *F*_1,38_ = 1.48, *P* = 0.2319). When experimental procedures were begun after four months of growth in the laboratory, females were larger than males (♂ 55.1 ± 0.43 vs. ♀ 59.7 ± 0.52 mm; F_1,36_ = 66.32, *P* < 0.0001). At the end of the second growth interval, female and male juveniles were ranked separately by SVL and were then assigned to size-matched treatment groups, as follows: (1) surgically castrated + placebo-implanted males (JM-CAST; *n* = 9; SVL: 55.3 ± 0.50 mm; range 53–57 mm); (2) surgically castrated + T-replaced males (JM-TEST; *n *= 9; SVL: 55.3 ± 0.60 mm; range 52–57 mm); (3) placebo-implanted control females (JF-CON; *n* = 10; SVL: 59.9 ± 0.66 mm; range 56–62 mm); and (4) T-supplemented females (JF-TEST; *n* = 10; SVL: 60.1 ± 0.57 mm; range 57–62 mm).

We anticipated that plasma T in juvenile males could be quite variable at the time of surgical treatments, and that an interpretation of effects of castration alone would therefore be ambiguous (see Cox and John-Alder 2005). Since our specific goal here was to investigate effects of T on IGF-1, we elected to “homogenize” the starting point of the experiment by castrating all of the juvenile males and replacing T in half of them. This experimental design created a more uniform starting point for all males while fully controlling replacement of T in castrated males, thus strengthening our specific focus on the effects of T. The experiment on juveniles was terminated after 36 days of treatment, which was sufficient time to measure significant differences in growth rate among treatment groups.

### T implants

Implants were constructed from 5 mm lengths of Silastic^®^ tubing (Dow Corning, Midland, MI) as previously described (Cox et al. 2005). After sealing one end of each tubule with silicone adhesive gel (Dow Corning), we used a Hamilton^®^ syringe to inject 3 µL of a solution of T dissolved in dimethyl sulfoxide (DMSO; 100 µg T/µL) into the open end. Each tubule was then sealed with silicone adhesive and stored at room temperature for several days to allow the DMSO to diffuse through the tubing and evaporate, leaving 300 µg of crystalline T within the lumen of each implant. Placebo implants were constructed in an identical fashion but were injected only with pure DMSO.

### Surgical treatments

Animals were anesthetized with an intramuscular injection of ketamine (200 mg/kg body mass; Vetus Animal Health, MFA Inc., Columbia, MO). In castrated and T-replaced males, the testes were exposed with a single ventral midline incision. Each spermatic cord was ligated with surgical silk, the testes were ablated, and the ligated spermatic cords were cauterized. Sham surgeries were done on control males and all groups of females. A T-filled or a placebo implant was inserted into the coelomic cavity, and the incision was closed with Surgi-Lock 2oc^™^ instant tissue adhesive (Meridian Animal Health, Omaha, NE).

### RNA isolation, RT-PCR, and qRT-PCR

At the conclusion of the treatment periods, lizards (*n* = 9 per treatment group) were killed by rapid decapitation, and liver tissue was harvested, flash frozen on dry ice, and stored at −80°C until analysis. Liver tissue was homogenized with TRIzol^®^ (Invitrogen—Thermo Fisher Scientific, Waltham, MA) using a Teflon pestle and a glass homogenizing tube, and total RNA was isolated according to the manufacturer’s protocol using Qiagen RNeasy Mini Kit (Qiagen Inc., Valencia, CA) coupled with DNase digestion. RNA purity and integrity were assessed by determination of the A_260_/A_280_ ratio and visualization of 28S and 18S ribosomal bands after electrophoresis on a 1% formaldehyde denaturing gel, respectively. RNA concentration was determined using the NanoDrop^®^ ND-1000 (Thermo Fisher Scientific Inc., Waltham, MA).

RNA (2 µg) was reverse-transcribed using the High-Capacity Reverse Transcription Kit (Thermo Fisher Scientific). Subsequently, gene-specific primers (*IGF-1*: 5′-TTGGTGGATGCTCTTCAGTTTG-3′/3′-CAGGTCACAGCTTTGGAAACAA-5′; *β-actin*: 5′-GAAGAGGAAGCAGCTGTGGC-3′/3′-GCTATGTTGCCTTGGACTTCG-5′), which were previously validated for *S. undulatus* (Duncan et al. 2015), were used to amplify 135 and 52 bp PCR products, respectively. All samples (*n* = 9 per treatment group) were run in triplicate, cDNA was fluoresced with SYBR^®^ Green (Applied Biosystems Inc.), and the threshold cycle (C_T_) of each sample was recorded on an ABI 7900HT. Standard curves of pooled cDNA were serially diluted so that the curve ranged from 250 ng to 25 pg. Within each experiment (i.e., adults versus juveniles), all samples in triplicate plus standard curves for *IGF-1* and *β-actin* were run in a single plate. Amplification efficiency of standard curves ranged from 95% to 100%, and *R*^2^ values exceeded 0.99 for all standard curves. Relative quantification of *IGF-1* was normalized to *β-actin*, which was unaffected by treatment (*F*_3,36_ = 0.42, *P* = 0.7390), and then compared to a calibrator (i.e., pooled RNA from experimental animals). The appropriate negative controls were run (i.e., No-RT control and master mix of reagents without template) to ensure the absence of DNA and non-specific amplification. The presence of a specific product was verified by visually inspecting the dissociation curves for each sample.

### Hormone assays

At the conclusion of the experimental periods, blood was collected from the post-orbital sinus as well as the neck and trunk wounds using heparinized microcapillary tubes (Fisher Scientific, Pittsburgh, PA). Blood collection was completed within 3 min to avoid prolonged stress. Plasma was separated via centrifugation and stored at −80°C until used for radioimmunoassay (RIA) for IGF-1. We did not have sufficient plasma to assay T in the present experiments. However, we have previously shown that surgical castration reduces plasma T to basal levels characteristic of females, and that implants such as those used here consistently restore plasma T to an average of ∼35 ng/mL, which is typical of breeding males (Cox et al. 2005; Cox and John-Alder 2005). Thus, treatment differences in T levels have previously been verified.

Assays for IGF-1 were conducted in G. Grau’s laboratory at the Hawaii Institute of Marine Biology, University of Hawaii. For RIA of IGF-1, proteins were extracted from plasma with acid-ethanol followed by cryoprecipitation to separate IGF-1 peptide from the IGF-binding proteins (IGFBPs) (Shimizu et al. 1999). Reconstituted samples were assayed in duplicate with ^125^I-recombinant salmon/trout (*Oncorhynchus* sp.) IGF-1 and rabbit anti-recombinant barramundi (*Lates calcarifer*) IGF-1 as the antiserum (Novozymes Biopharma AU Ltd., Adelaide, SA, AUS). The hormone complexes bound to antibody were precipitated from free radiolabel by the addition of goat anti-rabbit IgG (Sigma-Aldrich, St Louis, MO). This assay was previously validated for *S. undulatus* by demonstrating parallel binding to anti-recombinant barramundi IGF-1 in *S. undulatus* plasma (pool from intact *S. undulatus*) compared to a salmon/trout standard (Novozymes Biopharma AU Ltd.) and *O. mossambicus* plasma (Duncan 2011). The intra-assay variation, based on four aliquots of *S. undulatus* pooled plasma included in each assay, was 16% for adults and 4% for juveniles.

### Statistical analyses

All statistical analyses were performed using SAS versions 9.3 and 9.4 (SAS Institute Inc., Cary, NC, USA). For all response variables, treatment effects were initially analyzed in generalized linear models using SAS proc GLM, with body size (SVL at beginning of measurement period) and feeding rate (crickets/day) as covariates and with full interaction. To linearize relations between variables, body size (SVL), growth rate, and feeding rate were log-transformed for these analyses. Covariates and interaction terms were retained in final statistical models only when they were statistically significant at *α* = 0.05. On this basis, none of the final statistical models retained SVL or interactions with SVL as significant effects. Expression data for *IGF-1* mRNA were log-transformed prior to analysis to normalize their distribution.

For analyses restricted to adults, males and females were analyzed separately with treatment as the main effect because the number and identity of treatment groups were not balanced. Similarly, for analyses restricted to juveniles, males and females were initially analyzed separately because of unmatched treatment groups. Second, juveniles were analyzed using castrated males as the counterpart of female controls, allowing sex and treatment (placebo or T implant) to be entered as main effects. We recognize that female controls and male castrates are not comparable *sensu stricto*. However, by treating these groups as statistical counterparts, we could conduct a balanced comparison of sexes, and in no case did the outcomes differ qualitatively from separate analyses within sexes. Intact adult males were excluded from the analysis of the effect of age on IGF-1 hormone because this treatment was not represented in juveniles. All analyses were via one- or two-way analysis of variance (ANOVA) or analysis of covariance (ANCOVA) models using SAS proc GLM, with feeding rate entered as a covariate where appropriate. *Post hoc* comparisons were done via Ryan–Einot–Gabriel–Welsch test (REGWQ; SAS Institute 2002) in ANOVAs or by comparing least square means (lsmeans) in ANCOVAs. The Dunn–Sidàk correction for family-wise error was applied where appropriate.

## Results

### Growth and food consumption

#### Adults

Based on their body sizes (SVLs) at the time of capture, all adults in this study were at least 2 years of age and in their asymptotic growth phase (see Haenel and John-Alder 2002). Consistent with this determination, measurable growth did not occur in any of the adult treatment groups during the 14-day experiment, and experimental treatments had no discernible effects on growth.

Experimental treatments did not affect food consumption in adults (Table 1). Feeding rates (crickets/day) were identical in female groups (F-CON = 3.0 ± 0.01, F-TEST = 3.0 ± 0.01; *F*_1,16_ = 0.00; *P* = 1) and very similar among male groups (M-CON = 3.0 ± 0.00, M-CAST = 2.9 ± 0.04, M-TEST = 2.9 ± 0.03). Due to zero variance in M-CON, the slight differences in feeding rates achieved statistical significance in males (*F*_2,24_ = 5.43, *P* = 0.0114) due solely to the REGWQ contrast between M-CON and M-CAST. However, almost all of the reduced food consumption in M-CAST occurred during the first 6 days after surgery. During the final 8 days of the experimental period, only one male failed to eat all of his crickets, eating 23 of the 24 offered. Thus, the small reduction in food consumption in M-CAST is a result of surgery itself rather than a treatment effect.

**Table 1:** Feeding rate (crickets/day) for adults and juveniles

Treatment	Feeding rate (mean ± SE)	
	Adults	Juveniles
Male intact (M-CON)	3 ± 0	–
Male castrate (M-CAST)	2.87 ± 0.04	1.98 ± 0.08
Male T (M-TEST)	2.92 ± 0.03	1.84 ± 0.09
Female intact (F-CON)	2.99 ± 0.01	2.84 ± 0.05
Female T (F-TEST)	2.99 ± 0.01	2.07 ± 0.05

Adults: feeding rate was unaffected by treatments or sex and nearly the same in all groups. Juveniles: feeding rate was reduced by T in both sexes and lower in males than in females; the effect of T was greater in females than in males. Sample sizes: *n* = 9 in all adult groups and both juvenile male groups, *n* = 10 in juvenile female groups. See Results section “Juveniles” under the heading Growth and food consumption for details of statistical analyses. NB: feeding rate is not directly comparable between adults and juveniles because different-sized crickets were fed to the two age classes.

#### Juveniles

**Fig. 1:**
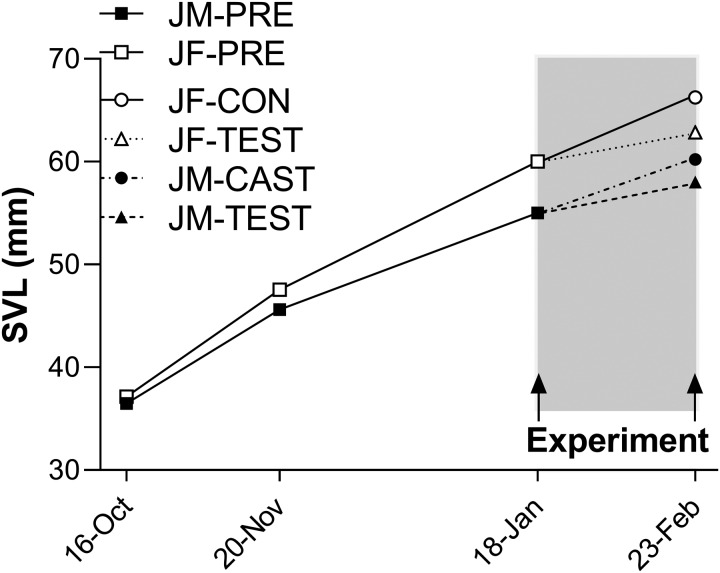
Body size of juvenile *S. undulatus* measured at three time points prior (JM-PRE and JF-PRE) and at one point after (JM-CAST, JM-TEST, JF-CON, and JF-TEST) experimental treatments. Growth rate is given by the slopes of the connecting lines in the two pre-experimental intervals and during the experiment itself (shaded region); error bars are omitted for clarity of presentation. Prior to treatments (square symbols), intact females grew faster than intact males to become ∼8% larger than males at the beginning of the experiment. During the experimental period, exogenous T significantly reduced growth rate to the same extent in females and in castrated males. See Results section “Juveniles” under the heading Growth and food consumption for statistical details.

In juveniles prior to experimental manipulations, growth rate (mm/day) was higher in females than in males and declined in both sexes as juveniles increased in size (Fig. 1). Females grew faster than males during the first (0.29 ± 0.011 vs. 0.26 ± 0.009 mm/day; *P* = 0.0065) and second (0.21 ± 0.007 vs. 0.16 ± 0.006 mm/day; *P* < 0.0001) growth intervals, and had become 8% larger than males by the beginning of the experimental period (60.0 ± 0.42 vs. 55.0 ± 0.42 mm; *P* < 0.0001).

None of the experimental groups of juveniles ate its entire daily ration of three crickets, indicating that juveniles were fed to satiety. Feeding rate differed significantly among groups (Table 1; *F*_3,34_ = 44.89, *P* < 0.0001). Feeding rate was higher in females than in males (*F*_1,34_ = 65.03, *P* < 0.0001) and was reduced by T (*F*_1,34_ = 44.96, *P* < 0.0001). Additionally, the significant treatment-by-sex interaction indicates that T reduced feeding rate to a greater extent in females than in males (*F*_1,34_ = 21.25, *P* < 0.0001).

**Fig. 2:**
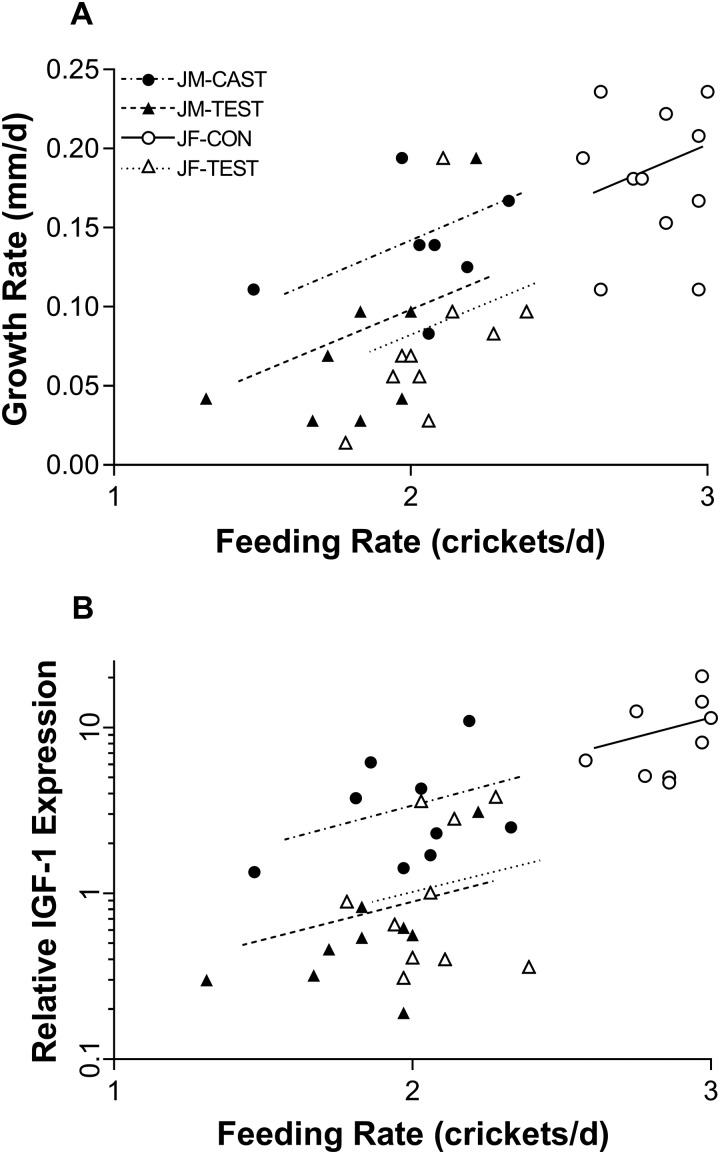
(**A**) Growth rate plotted versus feeding rate (crickets per day) in experimentally treated juvenile *S. undulatus*; *n* = 10 in females, *n* = 9 in males. Growth rate was significantly decreased by T in juveniles of both sexes after accounting for the effect of feeding rate. See Results section “Juveniles” under the heading Growth and food consumption for statistical details. (**B**) Relative expression of hepatic *IGF-1* mRNA plotted versus feeding rate in experimentally treated juvenile *S. undulatus*. Expression increased with increasing feeding rate and was significantly reduced by T in females and in castrated males. Symbols and lines as in Panel A; *n* = 9 in all groups. See Results section “Juveniles” under Hepatic *IGF-1* mRNA and plasma IGF-1for statistical details.

During the experimental period (shaded interval in Fig. 1), growth rate was significantly dependent on feeding rate and experimental treatment (overall ANCOVA; *F*_3,34_ = 15.22, *P* < 0.0001; Fig. 2A). In this model, growth rate increased with increasing feeding rate (*F*_1,34_ = 9.21, *P* = 0.0046) and was reduced by T both in females (JF-TEST) and in castrated males (JM-TEST) (*F*_1,34_ = 6.67, *P* = 0.0143) after accounting for differences in food intake, but sex itself did not have a significant effect on growth rate during the experimental period (*F*_1,34_ = 2.27; *P* = 0.1412). Interactions between treatment, sex, and feeding rate were not statistically significant and were therefore excluded from the final ANCOVA model.

Given that growth rate was significantly higher in intact females than in males prior to experimental treatments (above), it is particularly noteworthy that during the experimental period, growth rate did not differ significantly between castrated males and control females. Thus, castration of males eliminated but did not reverse the sex difference in growth rate that was evident prior to experimental treatments. At the same time, castrated males ate fewer crickets than control females and thus were somewhat more efficient than control females in terms of converting food into growth.

### Hepatic *IGF-1* mRNA and plasma IGF-1

#### Adults

**Fig. 3:**
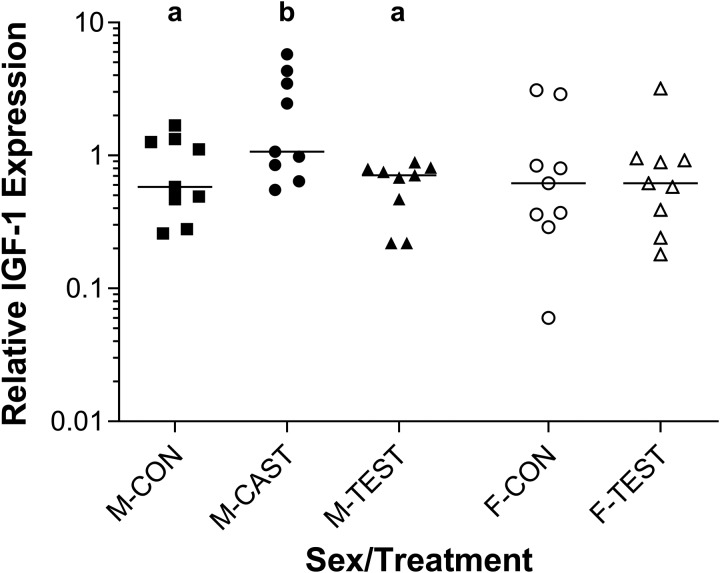
Relative expression of hepatic *IGF-1* mRNA in adult *S. undulatus*; *n* = 9 in all groups in adult males, mRNA expression was significantly increased by castration and returned to control levels by T replacement. In adult females, exogenous T had no significant effect. See Results section “Adults”under Hepatic *IGF-1* mRNA and plasma IGF-1 for statistical details.

In adult males, the relative expression of hepatic *IGF-1* mRNA was significantly increased by surgical castration and restored to control levels by T replacement (Fig. 3). After the 14-day treatment period, hepatic *IGF-1* mRNA was 3-fold higher in M-CAST than in M-CON and M-TEST (*F*_2,24_ = 5.34, *P* = 0.0121). In adult females (F-CON), relative hepatic *IGF-1* mRNA was indistinguishable from that of adult males (M-CON) (*F*_1,25_ = 0.74; *P* = 0.3985), and supplementation with T had no effect on hepatic *IGF-1* mRNA (F-CON vs. F-TEST: F_1,16_ = 0.10, *P* = 0.7582; Fig. 3).

**Fig. 4:**
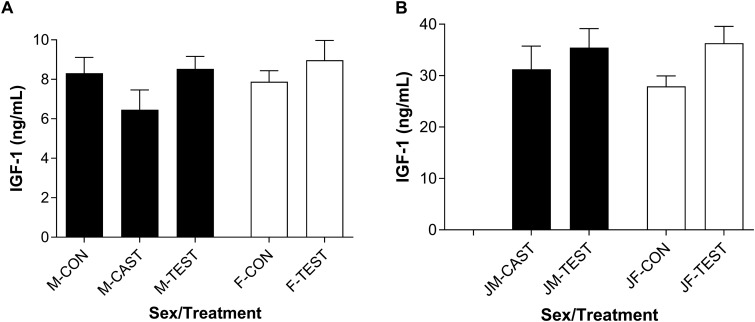
Plasma IGF-1 hormone in adult (**A**) and juvenile (**B**) *S. undulatus*. In both sexes, average IGF-1 concentrations were about 4× higher in juveniles than adults. Within either age group, IGF-1 did not differ between sexes and was not significantly affected by T. Columns represent the group mean ± 1 SE. Sample sizes: *n* = 9 in all groups except JF-CON, in which *n* = 10. See Results sections “Adults” and “Juveniles” under Hepatic *IGF-1* mRNA and plasma IGF-1 for statistical details.

In adults, plasma IGF-1 was not significantly affected by experimental treatments and did not differ between control males and control females (M-CON vs. F-CON: *F*_1,16_ = 1.53, *P* = 0.2345; Fig. 4A). In males, plasma IGF-1 tended to be reduced by castration and restored by T replacement, and in intact females, plasma IGF-1 tended to be increased by supplementation with T. However, none of these differences achieved statistical significance (adult males: *F*_2,24_ = 1.91, *P* = 0.1698; adult females: *F*_1,16_ = 0.92, *P* = 0.3514). Furthermore, an overall analysis of all adult treatment groups with relative *IGF-1* mRNA expression and treatment entered as main effects, only 14% of the variation in plasma IGF-1 hormone was explained by the full model (i.e., *R*^2^ = 0.14; *F*_5,39_ = 1.24, P = 0.3071).

#### Juveniles

In juveniles, the expression of hepatic *IGF-1* mRNA was significantly reduced by exogenous T (Fig. 2B). Hepatic *IGF-1* was strongly predicted by the model including sex and treatment as main effects and feeding rate as a covariate (*F*_3,33_ = 27.69, *P* < 0.0001; *R*^2^ = 0.715), although sex itself did not contribute significantly (*F*_1,33_ = 0.33, *P* = 0.5701). The expression of hepatic *IGF-1* mRNA was positively affected by feeding rate (*F*_1,33_ = 5.67, *P* = 0.0232) and was significantly reduced by T in females and in castrated males (*F*_1,33_ = 22.98, *P* < 0.0001).

Plasma IGF-1 was about four times higher in juveniles than in adults (∼30 vs. 8 ng IGF-1/mL; Fig. 4), and all comparisons of plasma IGF-1 between matched treatment groups of adults versus juveniles were highly significant at *P* < 0.0001. Plasma IGF-1 did not differ between JF-CON vs. JM-CAST: *F*_1,17_ = 0.49, *P* = 0.4927; Fig. 4B. As in adults, plasma IGF-1 was not significantly affected by experimental treatments in juveniles. In castrated juvenile males, plasma IGF-1 tended to be slightly increased by replacement of T (JM-CAST vs. JM-TEST), and similarly, in intact females, plasma IGF-1 tended to be increased by supplementation with T (JF-CON vs. JF-TEST), but these differences failed to achieve statistical significance (juvenile males: *F*_1,16_ = 0.52, *P* = 0.4794; juvenile females: *F*_1,18_ = 4.81; *P* = 0.0416). To achieve overall “family-wise” significance at *α* = 0.05 in these four comparisons, we applied the Dunn–Sidàk correction, which yielded *α* = 0.0127 as the acceptance level for any one comparison.

## Discussion

Previous research has demonstrated that T can inhibit growth in female-larger species and stimulate growth in male-larger species of lizards (Cox et al. 2005; Cox and John-Alder 2005; Cox et al. 2009; 2015), but the underlying mechanisms of this regulatory bipotentiality are not known. In species where it stimulates growth, T enhances functional outputs of the somatotrophic axis, including the expression of genes for hepatic *IGF-1* and peripheral IGF-1 receptors (*IGFr*; Borski et al. 1996; Riley et al. 2002; Larsen et al. 2004; Norbeck and Sheridan 2011; Cox et al. 2017), but how might T affect the somatotrophic axis in species where T inhibits growth? The novel results we report here begin to answer this question. In two independent experiments, we found that T inhibits the expression of hepatic *IGF-1* mRNA in *S. undulatus* (Eastern fence lizard), a female-larger species in which T inhibits growth. We found this inhibitory effect in growing juveniles of both sexes and in adult males (not females), in which perceptible growth was lacking. In juveniles, reduced expression of hepatic *IGF-1* mRNA was accompanied by a reduction in growth rate of similar magnitude in both sexes. To our knowledge, this is the first report of T inhibiting the expression of hepatic *IGF-1* mRNA, and therefore our findings stand in contrast to the published body of work. We attribute our novel result to the use of a female-larger species in which T inhibits organismal growth, whereas the existing literature is heavily biased toward studies using species in which T stimulates growth. Our findings suggest a relatively simple evolutionary reversal in the molecular switching mechanism by which T regulates the expression of hepatic *IGF-1* mRNA and may thus have far-reaching implications regarding evolutionary lability in the endocrine regulation of the central vertebrate growth axis. However, we also found that while plasma levels of IGF-1 hormone were about 4-fold higher in growing juveniles than in adults, reductions in *IGF-1* mRNA expression were not accompanied by significant changes in plasma IGF-1, creating apparent discordance (see Beckman 2011) between the growth response and the hormone response to T. As discussed below, no known mechanism of growth inhibition in juveniles can be attributed to a decrease in *IGF-1* mRNA in the absence of a concordant decrease in IGF-1 hormone. Other components of the somatotrophic axis (e.g., IGF receptors and IGFBPs) and perhaps other molecular systems (e.g., mechanistic target of rapamycin (mTOR)) will have to be taken into account, and further research will be needed to reveal fully the cellular and molecular mechanism(s) by which T inhibits growth.

We cannot be certain of the mechanism by which T inhibits the expression of *IGF-1* mRNA, but our results are more consistent with an “evolutionary reversal hypothesis” than with alternatives. In the simplest case, the evolutionary reversal hypothesis can be envisioned as invoking the classical T signaling pathway, in which T binds to a nuclear receptor in the cytoplasm and is then translocated into the cell nucleus, where it interacts with androgen response elements to influence gene expression (see Jenster et al. 1997; Cox 2020). In the present model, this pathway would lead directly to inhibition of the expression of hepatic *IGF-1* mRNA, where the novel aspect here is the inhibitory rather than stimulatory effect of T on the expression of *IGF-1* mRNA. Reversals in genomic effects of steroid hormones can evolve via several mechanisms, which may be as simple as point mutations in hormone response elements (Cox 2020) or alterations in the expression of genes that code for response element binding proteins, which compete with a hormone’s nuclear receptor for binding to target DNA (Lisse et al. 2011). Due to the simplicity of these mechanisms, and given that effects of steroids are known to differ even between sexes of a single species, it should be anticipated that in comparisons across taxonomic lines, evolutionary reversals in hormonal effects will be common.

In contrast to the evolutionary reversal hypothesis, three alternative hypotheses for growth inhibition are not supported by available evidence. (1) Aromatization hypothesis: Given the highly conserved nature of the vertebrate somatotrophic axis and the preponderance of evidence showing that T can increase and E_2_ can decrease functional activity of the somatotrophic axis, even within the same species (Riley et al. 2004; Norbeck and Sheridan 2011), the inhibitory effects of T in female-larger species may first require that T be aromatized into E_2_. However, Pollock et al. (2017) found that non-aromatizable 5α-DHT inhibits growth as effectively as T in *S. undulatus*, suggesting a mechanism of growth inhibition mediated by androgen receptors and not requiring aromatization of T to E_2_. (2) Energetic trade-off hypothesis: The near-equivalence of the estimated energetic cost of experimental T-induced activity versus the energetic savings of T-inhibited growth in *S. undulatus* under semi-natural conditions led Cox et al. (2005) to suggest that growth inhibition by T in *S. undulatus* might reflect an energy allocation trade-off. In the present report, however, we found that females in captivity grew faster and became significantly larger than males prior to experimental treatments under laboratory conditions in which substantial sex differences in energy allocation to activity could not have occurred. An allocation trade-off may contribute to sex differences in growth and the development of SSD in the field, but present results indicate that a trade-off is clearly not necessary to effect higher growth rates in females. (3) Nutritional inhibition hypothesis: Functional activity of the somatotrophic axis can be strongly influenced by an animal’s nutritional status (Duan 1998; Moriyama et al. 2000; Beckman et al. 2004; Pierce et al. 2005; Pedroso et al. 2006), and in this study, exogenous T caused reductions both in food intake and in growth rate. Thus, the inhibitory effect of T on *IGF-1* mRNA expression may have been mediated through under-nutrition. However, two lines of evidence in the present report fail to support this hypothesis. First, in adult males, castration caused an increase and T-replacement a decrease in the expression of *IGF-1* mRNA without affecting food consumption. Second, in juveniles, T caused substantial inhibition of the expression of *IGF-1* mRNA in both males and females even after statistical removal of the significant effect of food intake (Fig. 2B). Furthermore, Duncan et al. (2015) reported that extreme food deprivation (i.e., zero ration) was needed to inhibit the expression of *IGF-1* mRNA in *S. undulatus* and that even a 67% reduction in food intake was without effect. In the present experiment, *ad libitum* food intake was voluntarily reduced in juveniles by only ∼14% in males and 28% in females, well below the threshold needed to inhibit the expression of *IGF-1* mRNA. Thus, in summary, current evidence suggests that the bipotentiality of T as a growth regulator derives primarily from evolutionary reversibility in its direct effects on the expression of growth-regulatory genes rather than from alternative mechanisms.

Our findings imply evolutionary lability in the effect of T on a key member of the growth-regulatory gene network involving a simple reversal in the switching mechanism. If supported by further investigation, our model would indicate that the intervening molecular mechanisms between T and growth have evolved not as a tightly constrained functional unit but instead with the potential for diversification (see Hau 2007; Cox 2020). Effects of T on other components of the growth-regulatory gene network in both female-larger and male-larger species remain to be investigated before this model can be fully evaluated. However, in male-larger *A. sagrei*, in which T stimulates growth, Cox et al. (2017) have shown that T causes increased expression of a family of male-biased growth-regulatory genes, which code for receptors, binding proteins, and mTOR in addition to the hormone peptides themselves. By analogy, if genes associated with the somatotrophic axis have evolved as a functional module with only the switching effect of T having the potential to be an “on” or an “off” signal, then T could decrease the expression of these genes in female-larger species just as easily as it increases their expression in male-larger species.

Reduced expression of hepatic *IGF-1* mRNA was not accompanied by corresponding changes in plasma IGF-1 hormone in adults or in juveniles. On one hand, this is not surprising, as the correlation between transcript and protein is often quite weak, especially in perturbed systems with reduced transcript expression (Lee et al. 2011; Vogel and Marcotte 2012). Furthermore, in a review of the literature, Beckman (2011) reported that relationships involving *IGF-1* mRNA, IGF-1 hormone, and growth are quite variable, especially when mRNA and hormone are measured at only a single time point. For example, following a transfer from seawater to freshwater, tilapia (*Oreochromis mossambicus*) experienced reduced growth accompanied by a decrease in hepatic *IGF-1* mRNA and an increase in plasma IGF-1 compared to their counterparts that remained in seawater (Riley et al. 2003). In a study with juvenile hybrid striped bass (*Morone chrysops* × *Morone saxatilis*), partial food restriction increased hepatic *IGF-1* mRNA by 82% while plasma IGF-1 and growth were substantially decreased compared to *ad libitum*-fed controls (Picha et al. 2006). Following a period of re-feeding, hepatic *IGF-1* mRNA decreased by 61% but plasma IGF-1 and growth increased. Circumstances where hepatic *IGF-1* mRNA and plasma IGF-1 do not correlate might also be due to alterations in mRNA stability, clearance rates, degradation pathways, or availability of IGF-1 to its receptor, and to sequestration of IGF-1 by IGFBPs.

Regardless of the mechanism for the discordant effects of T, the lack of significant change in plasma IGF-1 begs the question of how reduced expression of hepatic *IGF-1* mRNA is linked to reduced growth in juvenile *S. undulatus*. The answer may derive from the complexity of the IGF-1 signaling pathway itself. In circulation, IGF-1 forms complexes with IGFBPs at a greater affinity than that of the type-I IGF receptor (Kelley et al. 2002). Exogenous T may have up-regulated IGFBPs that sequestered IGF-1 hormone and made it less readily available to its receptor. Additionally, if expression of other genes in the growth-regulatory network, including genes coding for IGFr in addition to the hormone itself, responds to T as a functional module, as suggested by the stimulatory effects of T on these genes in *A. sagrei* (Cox et al. 2017), then in species such as *S. undulatus*, T may inhibit the expression not only of hepatic *IGF-1* mRNA itself but also of other growth-regulatory genes including *IGFr* and genes of the mTOR pathway. If so, then the effectiveness of IGF-1 could be attenuated by a decrease in available *IGFr*s in target tissues as well as by downstream effects meditated through mTOR. Indeed, C. Cox et al. (SICB conference program 2018) have reported preliminarily that exogenous T exerts an inhibitory effect that mimics developmental divergence in the expression of genes in the GH/IGF-1 and mTOR pathways in *S. undulatus*. Thus, reduced expression of hepatic *IGF-1* mRNA alone may serve as a reliable proxy for the expression of other growth-regulatory genes, and a decrease in *IGF-1r* and other genes may be a key to fully understanding the connection between reductions in hepatic *IGF-1* mRNA expression and organismal growth without significant changes in plasma IGF-1 hormone.

We cannot currently assess the generality of the inhibitory effect of T on the expression of hepatic *IGF-1* mRNA, but this mechanism might be widespread in other species in which T inhibits growth, including lizards (Abell 1998; Cox and John-Alder 2005), snakes (Crews et al. 1985; Lerner and Mason 2001), birds (Sockman et al. 2008), and mammals (Swanson 1967). However, even when only considering lizards, growth inhibition by T is unlikely to be the sole mechanism through which female-biased SSD is achieved. Evolutionary changes in growth regulation by estrogens and other sex-specific factors can be expected to play important roles as well as T (see Kubička et al. 2013; Starostová et al. 2013; Kubička et al. 2015). Differences in mechanisms would be reminiscent of the case in fishes, in which the dominant growth-regulatory roles of androgenic and estrogenic hormones differ among species. Interestingly, all of the squamate species in which ovarian hormones may have a greater role than androgens are Gekkotans, while species in which T has been shown to be either stimulatory or inhibitory are Iguanians. Extant species of Gekkotan and Iguanian lizards have evolved following an ancient and early basal split in squamate phylogeny (Pyron et al. 2013), suggesting ample time and phylogenetic distance for variations in hormonal regulation of gene expression—as well as other growth-regulatory mechanism—to have evolved.

In summary, our novel finding that T inhibits the expression of hepatic *IGF-1* mRNA begins to answer the question of how hormones can have bipotential capacities, including the bipotential capacity of T to stimulate growth in many species, while inhibiting growth in others. While we have focused here on T, analogous capacities for bipotentiality could be generalizable to the effects of other hormone systems. We attribute our finding to our use of a female-larger species in which T inhibits growth, which stands in contrast to other studies in a literature dominated by male-larger species in which T stimulates growth. The mechanism we propose would in its simplest form require only an evolutionary reversal in the switching effect of T on a conserved module of growth-regulatory genes, allowing T to be either an “on” or an “off” switch. We cannot currently evaluate the generality of our finding or the model it suggests. Further comparative transcriptomic studies on squamates and other groups of vertebrates will be required to clarify these issues.

## Supplementary Material

obaa036_Supplementary_Data
