# Supplementary material for: Testosterone Reduces Growth and Hepatic IGF-1 mRNA in a Female-Larger Lizard, Sceloporus undulatus: Evidence of an Evolutionary Reversal in Growth Regulation
Source: Integr Org Biol. 2020 Oct 28;2(1):obaa036. doi: 10.1093/iob/obaa036 (PMC7715992; doi:10.1093/iob/obaa036)
Supplement: obaa036_Supplementary_Data [file obaa036_supplementary_data.doc]

**Figure Legends**

Figure 1. Body size of juvenile *Sceloporus undulatus* measured at three time points prior (JM-PRE and JF-PRE) and at one point after (JM-CAST, JM-TEST, JF-CON, JF-TEST) experimental treatments. Growth rate is given by the slopes of the connecting lines in the two pre-experimental intervals and during the experiment itself (shaded region); error bars are omitted for clarity of presentation. Prior to treatments (square symbols), intact females grew faster than intact males to become about 8% larger than males at the beginning of the experiment. During the experimental period, exogenous testosterone significantly reduced growth rate to the same extent in females and in castrated males. See Results section 3.1.2 for statistical details.

Figure 2. Panel A. Growth rate plotted versus feeding rate (crickets per day) in experimentally treated juvenile *S. undulatus*; n=10 in females, n=9 in males. Growth rate was significantly decreased by testosterone in juveniles of both sexes after accounting for the effect of feeding rate. See Results section 3.1.2 for statistical details. Panel B. Relative expression of hepatic *IGF-1* mRNA plotted versus feeding rate in experimentally treated juvenile *S. undulatus*. Expression increased with increasing feeding rate and was significantly reduced by testosterone in females and in castrated males. Symbols and lines as in Panel A; n=9 in all groups. See Results section 3.2.2 for statistical details.

Figure 3. Relative expression of hepatic *IGF-1* mRNA in adult *S. undulatus*; n=9 in all groups In adult males, mRNA expression was significantly increased by castration and returned to control levels by testosterone replacement. In adult females, exogenous testosterone had no significant effect. See See Results section 3.2.1 for statistical details.

Figure 4. Plasma IGF-1 hormone in adult (panel A) and juvenile (panel B) *S. undulatus*. In both sexes, average IGF-1 concentrations were about 4X higher in juveniles than adults. Within either age group, IGF-1 did not differ between sexes and was not significantly affected by testosterone. Columns represent the group mean ± 1 SE. Sample sizes: n=9 in all groups except JF-CON, in which n=10. See Results sections 3.2.1 and 3.2.2 for statistical details.
